# Prevalence and risk factors of curable sexually transmitted and reproductive tract infections and malaria co-infection among pregnant women at antenatal care booking in Kenya, Malawi and Tanzania: a cross-sectional study of randomised controlled trial data

**DOI:** 10.1136/bmjph-2023-000501

**Published:** 2024-09-18

**Authors:** Georgia R Gore-Langton, Mwayiwawo Madanitsa, Hellen C Barsosio, Daniel T R Minja, Jacklin Mosha, Reginald A Kavishe, George Mtove, Samwel Gesase, Omari A Msemo, Simon Kariuki, Kephas Otieno, Kamija S Phiri, John P A Lusingu, Crispin Mukerebe, Alphaxard Manjurano, Pius Ikigo, Queen Saidi, Eric D Onyango, Christentze Schmiegelow, James Dodd, Jenny Hill, Helle Hansson, Michael Alifrangis, Julie Gutman, Patricia Jean Hunter, Nigel Klein, Ulla Ashorn, Asma Khalil, Matt Cairns, Feiko O ter Kuile, R Matthew Chico

**Affiliations:** 1 Department of Disease Control, London School of Hygiene & Tropical Medicine, London, UK; 2 School of Global and Public Health, Kamuzu University of Health Sciences, Blantyre, Malawi; 3 Department of Clinical Sciences, Academy of Medical Sciences, Malawi University of Science and Technology, Thyolo, Malawi; 4 Kenya Medical Research Institute, Center for Global Health Research, Kisumu, Kenya; 5 National Institute for Medical Research, Tanga Centre, Tanga, Tanzania; 6 National Institute for Medical Research, Mwanza Centre, Mwanza, Tanzania; 7 Kilimanjaro Clinical Research Institute and Kilimanjaro Christian Medical University College, Moshi, Tanzania; 8 Centre for Medical Parasitology, Department of Immunology and Microbiology, University of Copenhagen and Department of Infectious Diseases, Copenhagen University Hospital, Copenhagen, Denmark; 9 Department of Gynaecology and Obstetrics, Copenhagen University Hospital—North Zealand, Hilleroed, Denmark; 10 Department of Clinical Sciences, Liverpool School of Tropical Medicine, Liverpool, UK; 11 Malaria Branch, Division of Parasitic Diseases and Malaria, Centre for Global Health, Centers for Disease Control and Prevention, Atlanta, Georgia, USA; 12 Great Ormond Street Institute of Child Health, University College London, London, UK; 13 Centre for Child Adolescent and Maternal Health Research, Faculty of Medicine and Health Technology, Tampere University, Tampere, Finland; 14 Fetal Medicine Unit, Saint George’s Hospital, London, UK; 15 Department of Infectious Disease Epidemiology, Faculty of Epidemiology and Population Health, London School of Hygiene & Tropical Medicine, London, UK

**Keywords:** public health, epidemiology, prevalence

## Abstract

**Objectives:**

Malaria and curable sexually transmitted and reproductive tract infections (STIs/RTIs) are associated with adverse pregnancy outcomes. This study reports the prevalence and risk factors of curable STIs/RTIs, STI/RTI co-infection and STI/RTI and malaria co-infection among HIV-negative pregnant women at their first antenatal care visit in Kenya, Malawi and Tanzania.

**Methods:**

HIV-negative pregnant women of all gravidae (n=4680) were screened for syphilis with point-of-care tests and treated if positive. Separately, women provided blood samples (n=4569) for rapid plasma reagin (RPR) testing; positive cases were confirmation by *Treponema pallidum* particle agglutination (TPPA). Women also provided dried blood spots for batch testing of malaria by retrospective polymerase chain reaction (PCR (n=4226) methods. A randomly selected subgroup of women provided vaginal swabs for chlamydia, gonorrhoea and trichomoniasis testing by retrospective PCR batch testing (n=1431), and bacterial vaginosis diagnosis by Nugent scoring (n=1402).

**Results:**

Malaria prevalence was 14.6% (95% CI 13.6 to 15.7), 45.9% (43.4 to 48.4) of women were positive for at least one curable STI/RTI and 6.7% (5.5 to 8.1) were co-infected with malaria and a curable STI/RTI. Prevalence of individual STIs/RTIs ranged from 28.5% (26.2 to 30.9) for bacterial vaginosis to 14.5% (12.7 to 16.4) for trichomoniasis, 13.8% (12.1 to 15.7) for chlamydia, 2.7% (1.9 to 3.6) for gonorrhoea and 1.7% (1.4 to 2.2) for RPR/TPPA-confirmed syphilis. The prevalence of STI/RTI co-infection was 10.1% (8.7 to 11.8). Paucigravidae, at highest risk of malaria, were also at greater risk of having chlamydia, gonorrhoea and bacterial vaginosis than multigravidae.

**Conclusions:**

Of women infected with malaria, 49.0% also had a curable STI/RTI and one in five women with at least one STI/RTI were co-infected with more than one STI/RTI. Current antenatal interventions that address malaria and curable STIs/RTIs remain suboptimal. New approaches to preventing and managing these infections in pregnancy are urgently needed.

**Trial registration number:**

NCT03208179.

**WHAT IS ALREADY KNOWN ON THIS TOPIC**

**WHAT THIS STUDY ADDS**

- This study adds critical information on the prevalence of malaria and STIs/RTIs in three sub-Saharan African countries and highlights the frequency of co-infection.

**HOW THIS STUDY MIGHT AFFECT RESEARCH, PRACTICE OR POLICY**

- Apart from syphilis, the antenatal care visit package lacks interventions that effectively target malaria and curable STIs/RTIs.
- In the near-term, this may involve the use of sulfadoxine-pyrimethamine combined with a partner antimalarial therapy such as dihydroartemisinin-piperaquine.
- Pregnant women also need access to affordable, rapid and accurate point-of-care diagnostics for curable STI/RTIs early in pregnancy and vaccines are needed to protect against these infections in pregnancy.

## Background

 Intermittent preventive treatment in pregnancy (IPTp) with sulfadoxine-pyrimethamine (SP) is one of the interventions recommended by WHO for the prevention of adverse consequences attributable to malaria during pregnancy in areas of moderate to high transmission. Malaria during pregnancy is associated with increased risk of numerous adverse fetal, newborn (including spontaneous abortion, stillbirth, preterm birth, low birth weight, small-for-gestational age) and maternal outcomes (maternal anaemia and death).^1^,^3^ In some countries, it is policy to test all women for malaria with rapid diagnostic tests (RDT) at antenatal care (ANC) booking, while in other countries relying on the presentation of symptoms is national policy. Sexually transmitted and reproductive tract infections (STIs/RTIs) are associated with ectopic pregnancies, preterm birth, small-for-gestational age, low birth weight, neonatal death as well as risk of acquiring and transmitting HIV.^4^,^6^ The five most common STIs/RTIs are curable: syphilis (*Treponema pallidum*), chlamydia (*Chlamydia trachomatis*), gonorrhoea (*Neisseria gonorrhoea*), trichomoniasis (*Trichomonas vaginalis*) and bacterial vaginosis. Syphilis is particularly pernicious, trebling the risk of stillbirth^7^ and causing 1 in 10 stillbirths in sub-Saharan Africa.^8^ WHO recommends universal syphilis screening at ANC booking and treatment of cases with benzathine penicillin G.^9^ For the diagnosis and treatment of other curable STIs/RTIs, WHO recommends symptom-based management in low-resource settings where laboratory facilities and trained staff may be limited.^10 11^ Syndromic management has proven effective among men who are most often symptomatic; however, women are far more likely to be asymptomatic and, therefore, less likely to be diagnosed and treated.^12^,^16^

In Burkina Faso, the prevalence of malaria and curable STI/RTI co-infection at ANC has been reported at 12.9%. This was based on malaria diagnosis by microscopy, point-of-care tests for syphilis and chlamydia and Nugent scoring for bacterial vaginosis.^15^ A study in Zambia using rapid plasma reagin (RPR) with *T. pallidum* haemagglutination assay to confirm syphilis, polymerase chain reaction (PCR to diagnose malaria, chlamydia, gonorrhoea and trichomoniasis, and Nugent scoring for bacterial vaginosis, reported the prevalence of malaria and curable STI/RTI co-infection as 38.7%^17^; >70% of the STI/RTI cases were asymptomatic.^13^

Information on curable STIs/RTIs during pregnancy is scarce in sub-Saharan Africa given the asymptomatic nature of most infections and the absence of screening for many infections at ANC, apart from HIV and syphilis. Using gold standard diagnostics in three East African countries, we investigated curable STI/RTI prevalence and risk factors, including malaria and curable STI/RTI co-infection with the aim of documenting a more complete estimate of disease burden during pregnancy and informing the development of integrated packages of care to address them and reduce their associated adverse consequences for maternal and newborn health.

## Methods

### Study design, population and study site

Participants were enrolled in the IMPROVE (IMproving PRegnancy Outcomes with intermittent preVEntative treatment in Africa) trial (NCT03208179), a three-arm malaria chemoprevention randomised controlled trial conducted in areas of perennial malaria transmission in Kenya, Malawi and Tanzania between 29 March 2018 and 5 July 2019; main trial outcomes have been published elsewhere.^18^ Eligible women were between 16 and 28 gestational weeks as measured by ultrasound, HIV-negative and had not previously received SP during the current pregnancy. Recruitment stopped when 4680 participants had been randomly assigned, as per sample size calculations detailed in appendix 8 of the main trial paper.^18^ Participants were allocated to receive at each scheduled ANC visit IPTp with SP (n=1561), or dihydroartemisinin-piperaquine (n=1561) or dihydroartemisinin-piperaquine plus the addition of 2 g azithromycin as part of the first visit only (n=1558) (online supplemental table 1). IPTp-SP is a single-day course and was directly observed in all instances. In contrast, dihydroartemisinin-piperaquine is a 3-day course and azithromycin was a 2-day course. Women in these two groups had IPTp directly observed, but took the remaining tablets home for self-administration. Self-reported adherence was high, exceeding 97.5%, a value validated through random and unannounced home visits over the course of the trial, along with telephone calls to all participants on the second and third days of each course.

**Table 1:** Sociodemographic and health characteristics of participants tested for malaria and STIs/RTIs

Sociodemographic and health characteristics*		Number of participants tested by infection (malaria, STIs/RTIs)			
		MalariaN=4226 (90.3%)	SyphilisN=4569 (97.6%)	Chlamydia, gonorrhoea, trichomoniasisN=1431 (30.6%)	Bacterial vaginosisN=1402 (30.0%)
		N (%)	N (%)	N (%)	N (%)
Maternal age (years)	<25 years	2274 (53.8)	2432 (53.2)	763 (53.4)	744 (53.1)
	>25 years	1950 (46.2)	2137 (46.8)	668 (46.7)	657 (46.9)
Gravidity	Paucigravidae	2345 (55.7)	2523 (55.4)	783 (55.0)	774 (55.4)
	Multigravidae	1868 (44.3)	2033 (44.6)	642 (45.0)	623 (44.6)
Marital status	Single	507 (12.0)	566 (12.4)	176 (12.3)	174 (12.4)
	Married	3715 (88.0)	4001 (87.6)	1254 (87.7)	1227 (87.6)
Socio-economic status by tercile	Low	1434 (34.0)	1516 (33.2)	466 (32.6)	440 (31.4)
	Medium	1417 (33.6)	1525 (33.4)	485 (33.9)	474 (33.8)
	High	1373 (32.5)	1528 (33.4)	480 (33.5)	488 (34.8)
Maternal education	None/Primary	2739 (65.0)	2963 (65.0)	900 (63.0)	869 (62.1)
	Secondary	1477 (35.0)	1598 (35.0)	528 (37.0)	530 (37.9)
Residency	Rural	3155 (74.7)	3351 (73.4)	1045 (73.1)	1026 (73.2)
	Semi-urban/Urban	1067 (25.3)	1216 (26.6)	385 (26.9)	375 (26.8)
Anaemia	No	2327 (55.3)	2535 (55.7)	777 (54.4)	766 (54.8)
	Yes	1882 (44.7)	2017 (44.3)	651 (45.6)	633 (45.3)

* The number of missing values was <5 for all socio-demographic and health characteristics.

RTIreproductive tract infectionSTIsexually transmitted infection

### Malaria and STI/RTI diagnosis

At enrolment, participants provided sociodemographic and maternal health information. Clinical staff collected a finger-prick blood sample from participants for retrospective batch analysis of malaria by ultrasensitive quantitative real-time-PCR.^19 20^ In Kenya and Tanzania, in keeping with national policy, all women were screened for malaria by RDT at enrolment and given first-line malaria treatment if positive. All women were screened for syphilis with a rapid point-of-care test (SD Bioline V.3.0). Positive cases were treated with 2.4 million units of benzathine penicillin G by intramuscular injection. As part of routine care, clinic staff queried women about whether they had experienced any symptoms associated with STIs/RTIs. Any suspected cases were assessed and treated by the clinic staff based on syndromic management guidelines.

Apart from routine care, all women provided venous blood for RPR (Plasmatec RPR Test Kit) analysis and syphilis confirmation by TPPA (Serodia). Additionally, clinic staff collected cervicovaginal swabs from a randomly selected subset of one-third of participants at each study site. Samples were shipped to a regional reference laboratory at the National Institute for Medical Research, Mwanza, Tanzania, at the end of the trial for retrospective batch analysis and, therefore, the test results were not used for clinical management. Samples were tested for *C. trachomatis* and *N. gonorrhoea* by RT-PCR (Artus CT/NG QS-RGQ Kit), trichomoniasis with SACASETM Real-TM Kit and bacterial vaginosis using the Nugent scoring (a case defined as a Nugent score of 7–10). Hereafter, when referring to curable STIs/RTIs, we specifically mean: syphilis, chlamydia, gonorrhoea, trichomoniasis and bacterial vaginosis.

### Data management and statistical analyses

Binary positive/negative outcome variables were generated for the five STIs/RTIs and malaria. When calculating the prevalence of at least one curable STI/RTI, analysis was restricted to the subset of participants who provided vaginal swabs to diagnose curable STIs/RTIs. We tested associations between maternal age (dichotomised around the median age; <25 years vs ≥25 years) and being positive for each individual STI/RTI using modified Poisson regression models to obtain prevalence ratios (PRs). We pooled data across the three countries, controlling for country and study site. Each potential explanatory variable: gravidity (paucigravidae (primigravidae and secundigravidae combined), multigravidae), maternal education (none/primary, secondary), marital status (single, married), residency (urban, rural), socio-economic status (SES) (low, medium, high tercile) and maternal anaemia (<11 g/dL), was added one-by-one to the model including maternal age, country and site. Being positive for bacterial vaginosis was also investigated as a potential risk factor for each of the four STIs. An explanatory variable which changed the PR of the STI/RTI by maternal age, country and site by >10% was included in the final multivariable model for that specific STI/RTI. Analyses were done in Stata (V.16, College Station, Texas, USA) and R (V.4.03).

We generated prevalence estimates of co-infection (positive for more than one STI/RTI or for malaria and at least one STI/RTI) and summarised co-infection combinations. Risk factors for the most common STI/RTI co-infection combinations were tested using multinomial logistic regression models to estimate the effect of explanatory variables on the probability that the outcome is in a particular category. For these models, the outcome measure was a mutually exclusive, multicategorical infection status variable, for example: (1) negative for bacterial vaginosis and trichomoniasis, (2) mono-infection with bacterial vaginosis, (3) mono-infection with trichomoniasis or (4) co-infection with bacterial vaginosis and trichomoniasis.

We present results first for individual infections: prevalence of malaria and then each STI/RTI, followed by risk factors for each STI/RTI. Prevalence of STI/RTI co-infection combinations is then presented followed by risk factors for the most common STI/RTI co-infection combinations. Finally, malaria and curable STI/RTI co-infection prevalence and risk factors are presented.

## Results

### Study population

In total, 4680 women were recruited, of whom 97.6% (n=4569) were tested for syphilis (RPR and TPPA) and 90.3% (n=4226) were tested for malaria by PCR. Randomly selected participants, 30.6% (n=1431), provided samples for gonorrhoea, chlamydia and trichomoniasis testing by PCR, and 30.0% (n=1402) for bacterial vaginosis testing by Nugent score. In total, 31.8% (n=1488) of women were tested for at least one of chlamydia, gonorrhoea, trichomoniasis or bacterial vaginosis. Maternal demographics did not differ by STI/RTI tested (table 1). See online supplemental figure 1 and online supplemental table 2 for further details on the number and combination of curable STIs/RTIs tested for.

### Prevalence: malaria and individual STIs/RTIs

Prevalence of malaria by PCR was 14.6% (95% CI 13.6 to 15.7) at enrolment. In total 469 women, 10.3% (9.4 to 11.2), were RPR positive, 79 of whom were confirmed by TPPA, giving a syphilis prevalence of 1.7% (1.4 to 2.2) (table 2) (see online supplemental table 3 for syphilis titre information). Among the subgroup tested, the prevalence of chlamydia was 13.8% (12.1 to 15.7), gonorrhoea 2.7% (1.9 to 3.6) and trichomoniasis 14.5% (12.7 to 16.4) (table 2, online supplemental figure 2). In Malawi, the prevalence of malaria (19.3% (17.2 to 21.5)) and syphilis (3.2% (2.4 to 4.3)) was higher than in Tanzania and Kenya. In Kenya, the prevalence of chlamydia was particularly high, at 21.0% (17.6 to 24.8) relative to Tanzania, 11.0% (8.7 to 13.8) and Malawi, 8.7% (6.2 to 12.2). In each of the three countries, bacterial vaginosis was the most common STI/RTI, with an overall prevalence of 28.5% (26.2 to 30.9). Among women with bacterial vaginosis, the mean Nugent score was 8.1; a further 9.8% (137/1402) of women had intermediary flora (Nugent score 4–6) (online supplemental table 3), among these women, 79.6% (109/137) were positive for clue cells. Among the subgroup tested, nearly one-half (45.9% (43.4 to 48.4); 683/1488)) were positive for at least one STI/RTI.

**Table 2:** Overall prevalence of malaria and STIs/RTIs at first ANC visit

	Total		Malawi		Tanzania		Kenya	
	Positive/Tested	Prevalence % (95% CI)	Positive/Tested	Prevalence % (95% CI)	Positive/Tested	Prevalence % (95% CI)	Positive/Tested	Prevalence % (95% CI)
Malaria	618/4226	14.6 (13.6 to 15.7)	250/1298	19.3 (17.2 to 21.5)	179/1628	11.0 (9.6 to 12.6)	189/1298	14.6 (12.7 to 16.6)
Syphilis	79/4569	1.7 (1.4 to 2.2)	42/1321	3.2 (2.4 to 4.3)	30/1772	1.7 (1.2 to 2.4)	7/1476	0.5 (0.2 to 1.0)
Chlamydia	198/1431	13.8 (12.1 to 15.7)	31/355	8.7 (6.2 to 12.2)	65/590	11.0 (8.7 to 13.8)	102/486	21.0 (17.6 to 24.8)
Gonorrhoea	38/1431	2.7 (1.9 to 3.6)	11/355	3.1 (1.7 to 5.5)	11/590	1.9 (1.0 to 3.3)	16/486	3.3 (2.0 to 5.3)
Trichomoniasis	207/1431	14.5 (12.7 to 16.4)	60/355	16.9 (13.4 to 21.2)	92/590	15.6 (12.9 to 18.8)	55/486	11.3 (8.8 to 14.5)
Bacterial vaginosis	399/1402	28.5 (26.2 to 30.9)	102/327	31.2 (26.4 to 36.4)	171/586	29.2 (25.6 to 33.0)	126/489	25.8 (22.1 to 29.8)
>1 STI/RTI	683/1488	45.9 (43.4 to 48.4)	163/391	41.7 (36.9 to 46.7)	283/593	47.7 (43.7 to 51.8)	237/504	47.0 (42.7 to 51.4)
>1 STI/RTI and malaria	94/1401	6.7 (5.5 to 8.1)	33/378	8.7 (6.3 to 12.0)	30/543	5.5 (3.9 to 7.8)	31/480	6.5 (4.6 to 9.0)

Note: Previous reporting of STI/RTI prevalence was restricted to a slightly smaller subgroup than presented here.^18^ These estimates account for all STI/RTI diagnostic results.

RTIreproductive tract infectionSTIsexually transmitted infection

**Table 3:** Risk factors for syphilis, chlamydia, gonorrhoea and bacterial vaginosis during pregnancy

STI/RTI	Risk factor		Positive	Tested	Prevalence % (95% CI)	Prevalence ratio (95% CI)	P value
Syphilis	Maternal age	<25 years	38	2432	1.6 (1.1 to 2.1)	1.4 (0.4 to 4.9)	0.578
		>25 years	41	2137	1.9 (1.4 to 2.6)	Ref	
	Gravidity	Paucigravidae	32	2523	1.3 (0.9 to 1.8)	0.5 (0.2 to 1.7)	0.274
		Multigravidae	47	2033	2.3 (1.7 to 3.1)	Ref	
	Socio-economic tercile	Low	44	1516	2.9 (2.2 to 3.9)	**8.8 (1.0 to 76.1**)	**0.048**
		**Medium**	**26**	1525	**1.7 (1.2 to 2.5**)	**8.8 (1.1 to 68.1**)	**0.037**
		High	9	1528	0.6 (0.3 to 1.1)	Ref	
	Bacterial vaginosis	Negative	9	987	0.9 (0.5,1.7)	Ref	
		**Positive**	**14**	**391**	**3.6 (2.1 to 6.0**)	**3.7 (1.6 to 8.4**)	**0.002**
Chlamydia	Maternal age	<25 years	124	763	16.3 (13.8 to 19.0)	1.0 (0.7 to 1.4)	0.955
		>25 years	74	668	11.1 (8.9 to 13.7)	Ref	
	Gravidity	**Paucigravidae**	**137**	**783**	**17.5 (15.0 to 20.3**)	**1.7 (1.2 to 2.4**)	**0.002**
		Multigravidae	61	642	9.5 (7.5 to 12.0)	Ref	
Gonorrhoea	Maternal age	<25 years	28	763	3.7 (2.5 to 5.3)	1.6 (0.7 to 3.3)	0.237
		>25 years	10	668	1.5 (0.8 to 2.8)	Ref	
	Gravidity	**Paucigravidae**	**29**	**783**	**3.7 (2.6 to 5.3**)	**2.5 (1.2 to 5.4**)	**0.018**
		Multigravidae	9	642	1.4 (0.7 to 2.7)	Ref	
	Maternal education	**None/Primary**	**27**	**900**	**3.0 (2.1 to 4.3**)	**2.4 (1.2 to 5.1**)	**0.016**
		Secondary/higher	11	528	2.1 (1.2 to 3.7)	Ref	
	Socio-economic tercile	Low	14	466	3.0 (1.8 to 5.0)	0.9 (0.3 to 2.6)	0.876
		Medium	11	485	2.3 (1.3 to 4.1)	0.5 (0.2 to 1.0)	0.054
		High	13	480	2.7 (1.5 to 4.6)	Ref	
	Bacterial vaginosis	Negative	21	962	2.2 (1.4 to 3.3)	Ref	
		Positive	16	383	4.2 (2.6 to 6.7)	1.8 (1.0 to 3.4)	0.074
Bacterial vaginosis	Maternal age	<25 years	222	744	29.8 (26.5 to 33.1)	1.0 (0.8 to 1.2)	0.752
		>25 years	177	658	26.9 (23.6 to 30.4)	Ref	
	Gravidity	**Paucigravidae**	**241**	**774**	**31.1 (27.8 to 34.4**)	**1.3 (1.1 to 1.6**)	**0.018**
		Multigravidae	156	623	25.0 (21.8 to 28.6)	Ref	

Country and study site controlled for in all models.

Statistically significant results are in bold.

RefreferenceRTIreproductive tract infectionSTIsexually transmitted infection

### Risk factors associated with individual STI/RTIs

Results of the final adjusted models with each individual STI/RTI as the outcome of interest, and the variables listed in the ‘Methods’ section explored as risk factors are shown in table 3 for syphilis, chlamydia, gonorrhoea and bacterial vaginosis. In the final syphilis multivariable model, being positive for bacterial vaginosis was associated with an increased risk of infection; the prevalence of syphilis was 3.6% among women with bacterial vaginosis and 0.9% among women without bacterial vaginosis (PR: 3.7; 95% CI 1.6 to 8.4), p=0.002 (table 3, online supplemental table 4). Women of low or medium SES were almost nine times more likely to have syphilis than women of high SES (table 3). Neither gravidity nor maternal age were associated with syphilis. Prevalence was slightly higher among multigravidae, 2.3% than paucigravidae, 1.3%.

Compared with multigravidae, paucigravidae were more likely to have chlamydia, gonorrhoea and bacterial vaginosis. When controlling for maternal age, chlamydia prevalence was 70% higher (PR 1.7; 95% CI 1.2 to 2.4, p=0.002) among paucigravidae than among multigravidae, and bacterial vaginosis was 30% higher (PR 1.3; 95% CI 1.1 to 1.6, p=0.018) (table 3, online supplemental tables 5 and 6). When controlling for maternal age and education level, the prevalence of gonorrhoea among paucigravidae women was more than double that of multigravidae (PR 2.5; 95% CI 1.2 to 5.4, p=0.018 (table 3, online supplemental table 7).

There were no subgroups identified as being at elevated risk of having trichomoniasis in either the univariate or the multivariate analyses (online supplemental table 8). While maternal age and gravidity were risk factors in univariate analyses for the composite outcome of at least one of chlamydia, gonorrhoea, trichomoniasis or bacterial vaginosis, these were not associated in the final multivariable model (online supplemental table 9).

### Prevalence: STI/RTI co-infection

Among the 678 women positive for an STI/RTI and tested for additional STIs/RTIs, 22.0% (n=149) were co-infected with at least one other STI/RTI, giving an overall co-infection prevalence of 10.1% (95% CI 8.7 to 11.8) among all women tested for at least two STIs/RTIs. Of co-infected women, 80.5% (n=120) had two STIs/RTIs, 16.8% (n=25) had three STIs/RTIs, 2.0% (n=3) had four STIs/RTIs and one woman (0.7%) was co-infected with all five curable STIs/RTIs.

**Figure 1:**
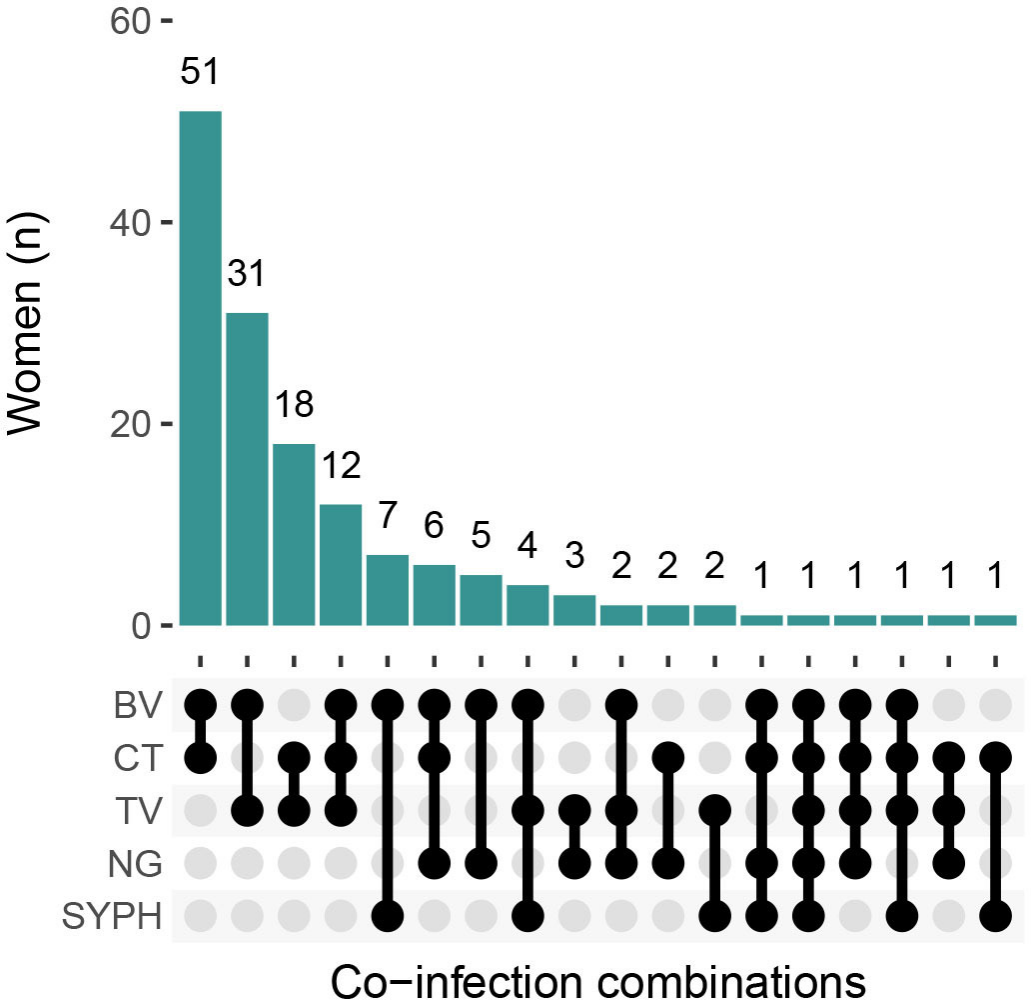
Frequency of unique combinations of sexually transmitted infection and reproductive tract infection co-infections. BV, bacterial vaginosis; CT, chlamydia; NG, gonorrhoea; SYPH, syphilis; TV, trichomoniasis;.

The most common unique co-infection combination was bacterial vaginosis and chlamydia (n=51) (a further 22 women were co-infected with these two and additional STIs/RTIs), followed by bacterial vaginosis and trichomoniasis (n=31) (a further 21 women were co-infected with these two plus additional STIs/RTIs), 18 women were co-infected with chlamydia and trichomoniasis (a further 16 women were co-infected with these two plus additional STIs/RTIs). The fourth most common co-infection combination was bacterial vaginosis, trichomoniasis and chlamydia (n=12) (figure 1).

For women positive for each STI/RTI, online supplemental table 10 shows the proportion co-infected with the other four curable STIs/RTIs. Notably, among women who had syphilis, 60.8% (14/23) also had bacterial vaginosis. Among women with gonorrhoea, 43.2% (16/37) were co-infected with bacterial vaginosis, 31.6% (12/38) with chlamydia and 21.1% (8/38) with trichomoniasis. Among women positive for chlamydia, 37.8% (73/193) also had bacterial vaginosis and 17.2% (34/198) also had trichomoniasis.

### Risk factors for STI/RTI co-infection

For the two most common STI/RTI co-infection combinations, primigravidae were nearly three times more likely than multigravidae to be co-infected with both STIs/RTIs than have neither infection (online supplemental table 11). Specifically, relative risk ratios (RRRs) of having a co-infection rather than neither infection among paucigravidae relative to multigravidae were RRR=2.6 (95% CI 1.2 to 5.7, p=0.020) for bacterial vaginosis and trichomoniasis, and RRR=2.9 (1.4 to 5.7, p=0.003) for bacterial vaginosis and chlamydia.

When controlling for all variables, women who were positive for bacterial vaginosis were 1.5 (95% CI 1.2 to 2.0 p=0.002) times more likely to have chlamydia, and 3.7 (95% CI 1.6 to 8.6, p=0.002) times more likely to have syphilis, relative to women without bacterial vaginosis (online supplemental table 12).

### Prevalence: STI/RTIs and malaria co-infection

Among the subgroup of women tested for either chlamydia, gonorrhoea, trichomoniasis or bacterial vaginosis at the first ANC visit, 1401 were also tested for malaria by PCR. Of these, 6.7% (95% CI 5.5 to 8.1, n=94) had malaria plus an STI/RTI co-infection, 39.3% (95% CI 36.8 to 41.9, n=551) had STI/RTI only, 7.0% (95% CI 5.8 to 8.5, n=98) had malaria only and 47.0% (95% CI 44.4 to 49.6, n=658) had neither malaria nor an STI/RTI; see online supplemental table 13 for the prevalence of specific co-infection combinations. Prevalence of malaria and a curable STI/RTI co-infection was similar among paucigravidae (7.1% (95% CI 5.5 to 9.2)) and multigravidae (6.2% (95% CI 4.6 to 8.4)), p=0.508. Among women positive for malaria and tested for chlamydia, gonorrhoea, trichomoniasis or bacterial vaginosis, one-half (49.0%, 95% CI 41.9 to 56.0; n=94/192) also had at least one STI/RTI. The prevalence of malaria co-infection among women with bacterial vaginosis did not differ by Nugent score nor did the prevalence of malaria co-infection among women with syphilis differ by RPR titre (online supplemental figures 3, 4 and online supplemental table 14).

### Risk factors for malaria and curable STI/RTI co-infection

Risk factor analysis of malaria and curable STI/RTI co-infection identified women who had anaemia as being at greater risk of having a malaria and curable STI/RTI co-infection, rather than neither infection, specifically malaria and chlamydia (PR 7.7 (95% CI 2.5 to 23.7), p<0.001); malaria and trichomoniasis (PR 3.5 (95% CI 1.3 to 9.6), p=0.013); malaria and bacterial vaginosis (PR 2.2 (95% CI 1.2 to 4.1), p=0.011) (online supplemental table 15).

## Discussion

Our analysis of malaria and STI/RTI prevalence and risk factors associated with either STI/RTI, STI/RTI co-infection or STI/RTI-malaria co-infections provides important insight into the epidemiology of infections in pregnancy in three East African countries. We report a very high prevalence of women with at least one curable STI/RTI (45.9%), highlighting the need for improved diagnosis and treatment interventions at ANC, where currently women are rarely screened, unless symptomatic.

The burden of malaria in pregnancy is reduced with the universal provision of IPTp with SP at scheduled ANC visits from the second trimester to delivery, although coverage remains inadequate and many malaria parasites have lost sensitivity to the intervention. Syphilis is managed with universal screening and treatment at ANC booking. Other curable STIs/RTIs, however, are relatively neglected. Presumptive treatment of all women for STIs/RTIs alongside IPTp has produced mixed results and raises important issues around antimicrobial resistance.^21 22^ The way forward in the near term for curable STIs/RTIs will likely involve options that range from universal screening for curable STIs/RTIs to risk-based presumptive treatment or risk-based screening-and-treatment.

Individual estimates of trichomoniasis and bacterial vaginosis prevalence were lower than recent estimates among pregnant women in East and Southern Africa.^17 23^ We found that 3 in 10 women had bacterial vaginosis, a consequential burden given its association with adverse reproductive and obstetric sequalae, and increased risk of STI and HIV acquisition and transmission.^24 25^ While prior reports of the prevalence of STI/RTI indicated a prevalence of chlamydia of 5.0% among the general population of women in the WHO African region^26^ and 5.2% among ANC attendees in Eastern and Southern Africa,^23^ we found a prevalence nearly three times higher at 13.8%. The prevalence was particularly high in Kenya, where one in five women overall, and one in four paucigravidae, had chlamydia. Our findings are in line with recent results among women 15–49 years of age in Zimbabwe^27^ and South Africa,^28^ potentially suggesting that the prevalence of chlamydia has increased in the subregion over the past decade. Antenatal services should aim to treat chlamydia infection early in pregnancy in order to prevent the associated adverse pregnancy outcomes.^29^ The importance of this cannot be overstated; a secondary analysis of data from our trial found that fetal growth was restricted in women who had a curable STI/RTI in the second trimester but was not restricted among women with an STI/RTI occurring only in the third trimester.^30^ Advancements in the development of rapid, simple and well-performing point-of-care tests for chlamydia and gonorrhoea are much needed.^31^

We previously reported that the prevalence of at least one STI/RTI was 16.9%.^18^ This reflected results from the entire study population, for whom two-thirds of women were only tested for syphilis. In this current analysis, we restricted data to the randomly selected subsample of women tested for chlamydia, gonorrhoea, trichomoniasis and bacterial vaginosis. The prevalence of having at least one STI/RTI among this subgroup who underwent comprehensive STI/RTI testing was three times higher, 45.9%.

Paucigravidae were at greatest risk of individual STI/RTI and STI/RTI co-infection, regardless of their age. Many reasons may contribute to this difference. Paucigravidae may have comparatively lower acquired immunity than multigravidae due to fewer lifetime exposures, and may thus harbour STIs/RTIs for longer. Given the association between STIs/RTIs and pelvic inflammatory disease and infertility,^32^ it is possible that women attending ANC as multigravidae (women able to conceive numerous times) would be those with a lower prevalence of STIs/RTIs.

More women had at least one curable STI/RTI in this population than had malaria,^18^ and yet no systematic interventions or screening strategies beyond syndromic management are in place, with the exception of syphilis screening (the coverage of which can also be suboptimal).^33 34^ Results of ongoing trials investigating the integration of curable STI/RTI screening and treatment interventions at ANC will provide much-needed data.^35 36^

The British HIV Association recommend screening HIV-infected pregnant women for bacterial vaginosis.^37^ Similarly, given that 60.9% of women with syphilis were positive for bacterial vaginosis, presumptive bacterial vaginosis treatment or a screen-and-treat approach could be considered among all women with a positive syphilis test result. However, the low syphilis prevalence (0.6%) in this setting would have meant that just 3.6% of bacterial vaginosis cases would have received enhanced care under this approach. The low syphilis prevalence may also explain the reason that known risk factors such as maternal age and gravidity were not found to be statistically associated here. Presumptive treatment or a screen-and-treat approach for bacterial vaginosis based on STI presence could be extended to all women with any STI since among those with chlamydia, gonorrhoea and trichomoniasis, 37.8%, 43.2% and 26.4%, respectively, also had bacterial vaginosis. However, this would first require introduction of point-of-care testing for these infections.

Understanding the epidemiology of curable STIs/RTIs associated with adverse pregnancy outcomes is especially important in areas of malaria transmission, as almost half of the women with malaria in our study were co-infected with a curable STI/RTI. A cohort study nested within the IMPROVE trial showed that infection during pregnancy with malaria-only, STI/RTI-only or co-infection with both, was associated with reduced fetal growth when compared with women without malaria or STI/RTIs. Malaria and STI/RTI co-infection had the most detrimental effect on fetal growth compared with malaria mono-infection or STIs/RTIs mono-infection.^30^ As clinical trials continue to investigate alternatives to SP for IPTp, collecting data on malaria in pregnancy and on curable STIs/RTIs and co-infection are needed should the non-malarial effects conferred by different IPTp regimens be fully appreciated and the benefits of IPTp on improving birth outcomes maximised.^38^

Strengths of this study include the large sample size that included sites in three East African countries, and the use of gold standard diagnostics for the five curable STIs/RTIs and malaria. HIV-infected women were excluded from the trial because WHO does not recommend providing IPTp with SP to women who are already receiving cotrimoxazole. SP and cotrimoxazole both contain sulfa and there is a theoretical risk of severe adverse reactions if taken concomitantly. Because the prevalence of malaria and STIs/RTIs among women living with HIV is higher than among HIV-negative women,^39 40^ our prevalence estimates likely understate the true burden among all pregnant women attending ANC.

In addition, 1041 women chose not the participate in the trial or their partner/spouse/another family member discouraged them from joining. We do not know if their non-participation affected our estimates. Finally, our prevalence and risk estimates cannot be assumed to be representative of all pregnant women; ANC attendees, and particularly those women who attended early enough to be eligible for inclusion in the trial, are not necessarily representative of the general pregnant population. Lack of ANC attendance and the opportunities it provides for interventions has been reported as a risk factor for curable STIs/RTIs.^41^ Our findings may not reflect the true burden of disease at the community level. Community delivery of IPTp is now a WHO recommendation to complement ANC IPTp administration. Further research into the burden of curable STIs/RTIs among non-ANC attending pregnant women is needed to inform interventions among this group.

In summary, malaria infection and curable STIs/RTIs were highly prevalent among women attending their first ANC visit, with paucigravidae at particular risk. Our results underscore the need for novel approaches to reduce the burden of malaria and curable STIs/RTIs in pregnancy and the need for further research into the burden of infection and co-infection, particularly given that improving pregnancy outcomes may be more difficult to achieve in the presence of co-infection without combination interventions. This will necessitate holistic approaches to ANC. In malaria-endemic East and Southern Africa, this may involve use of SP in combination with a more potent antimalarial therapy such as dihydroartemisinin-piperaquine alongside affordable, rapid and accurate point-of-care diagnostics for curable STIs/RTIs as early as possible in pregnancy.

## supplementary material

10.1136/bmjph-2023-000501 online supplemental file 1

10.1136/bmjph-2023-000501 online supplemental file 2

## Data Availability

Data are available on reasonable request.
