# Supplementary material for: Prevalence and risk factors of curable sexually transmitted and reproductive tract infections and malaria co-infection among pregnant women at antenatal care booking in Kenya, Malawi and Tanzania: a cross-sectional study of randomised controlled trial data
Source: BMJ Public Health. 2024 Sep 18;2(2):e000501. doi: 10.1136/bmjph-2023-000501 (PMC11816199; doi:10.1136/bmjph-2023-000501)
Supplement: online supplemental file 1 [file bmjph-2-2-s001.pdf]

## Supporting Information

# Co-infection prevalence and risk factors: malaria and curable sexually transmitted and reproductive tract infections at antenatal care booking in Kenya, Malawi, and Tanzania

Georgia Gore-Langton, Mwayiwawo Madanitsa, Hellen C. Barsosio, Daniel T R Minja, Jacklin F Mosha, Reginald A Kavishe, George Mtove, Samwel Gesase, Omari A Msemo, Simon Kariuki, Kephas Otieno, Kamija S Phiri, John P A Lusingu, Crispin Mukerebe, Alphaxard Manjurano, Pius Ikigo, Queen Saidi, Eric D Onyango, Christentze Schmiegelow, James Dodd, Jenny Hill, Helle Hansson, Michael Alifrangis, Julie R Gutman, Patricia Hunter, Nigel Klein, Ulla Ashorn, Asma Khalil, Matthew E Cairns, Feiko O ter Kuile, R Matthew Chico

Table of Contents

Table 1: IMPROVE intervention arms ........ 3

Figure 1: Unique STI/RTI test combinations and number tested ........ 4

Table 2: Total number of STI/RTI tests done per woman ........ 4

Table 3: Bacterial vaginosis and syphilis infection results ........ 5

Figure 2: Prevalence of each individual infection and prevalence of at least one of chlamydia, gonorrhoea, trichomoniasis, or bacterial vaginosis ........ 5

Table 4: Logistic regression models for association of risk factors with syphilis prevalence (all models controlled for country and site) ........ 6

Table 5: Logistic regression models for association of risk factors with chlamydia prevalence (all models controlled for country and site) ........ 7

Table 6: Logistic regression models for association of risk factors with bacterial vaginosis prevalence (all models controlled for country and site) ........ 8

Table 7: Logistic regression models for association of risk factors with gonorrhoea prevalence (all models controlled for country and site) ........ 9

Table 8: Logistic regression models for association of risk factors with trichomoniasis prevalence (all models controlled for country and site) ........ 10

Table 9: Logistic regression models for association of risk factors with at least one of CT/NG/TV/BV prevalence (all models controlled for country and site) ........ 11

Table 10: Proportion of women positive with each individual STI/RTI who are co-infected with each of the other four STI/RTIs ........ 12

Table 11: Multinomial logistic regression models for association of risk factors with the three most common co-infection combinations ........ 13

Table 12: Association between each STI/RTIs pair (crude and adjusted): ........ 14

Table 13: Prevalence of malaria and curable STI/RTI co-infection ........ 15

Figure 3: Number of TPPA confirmed syphilis cases by RPR titre (grey bar) and number co-infected with malaria (red bar) ........ 15

Figure 4: Number of BV cases by categorical Nugent score (grey bar) and number co-infected with malaria (red bar) ........ 16

Table 14: Number and proportion of women co-infected with malaria by categorical BV and syphilis status ........ 16

Table 15: Multinomial logistic regression models for association of risk factors with malaria and bacterial vaginosis/chlamydia/trichomoniasis co-infection. ........ 17

**Table 1: IMPROVE intervention arms**

| Arm | Drug regimen |
|---|---|
| **IPTp-SP** | This is the control arm and consists of a standard single-day stat course of 3 tablets of quality-assured SP (tablets of 500 mg of sulphadoxine and 25 mg of pyrimethamine) provided at enrolment and at each subsequent monthly ANC visit in the 2nd and 3rd trimester. |
| **IPTp-DP + Azithromycin placebo** | 3 to 5 tablets of DP (tablets of 40 mg of dihydroartemisinin and 320 mg of piperaquine, based on bodyweight) daily for 3 days, plus 1 tablet of placebo Azithromycin (AZ) daily for 2 days. The dose of DP is equivalent to the standard treatment (case-management) dose for malaria in adults recommended by WHO with a target dose (range) of 4 (2-10) mg/kg bodyweight per day dihydroartemisinin and 18 (16-27) mg/kg bodyweight per day piperaquine given once a day for 3 days for adults and children weighing>= 25 kg (36-<60 kg: 3 tablets, 60-<80 kg:4 tablets, >=80 kg 5 tablets). One 3-day course was provided at enrolment and again at each subsequent monthly ANC visit in the 2nd and 3rd trimester. The weight at enrolment was used to define the weight class to guide treatment both at enrolment and subsequent scheduled visits. |
| **IPTp-DP + Azithromycin** | 3 to 5 tablets of DP (based on bodyweight) daily for 3 days, plus two azithromycin tablets containing 500 given orally once daily for 2 consecutive days (cumulative dose of 2 g) at the same time as the first and second daily dose of DP at enrolment. DP course was provided at enrolment and again at each subsequent monthly ANC visit in the 2nd and 3rd trimester. |

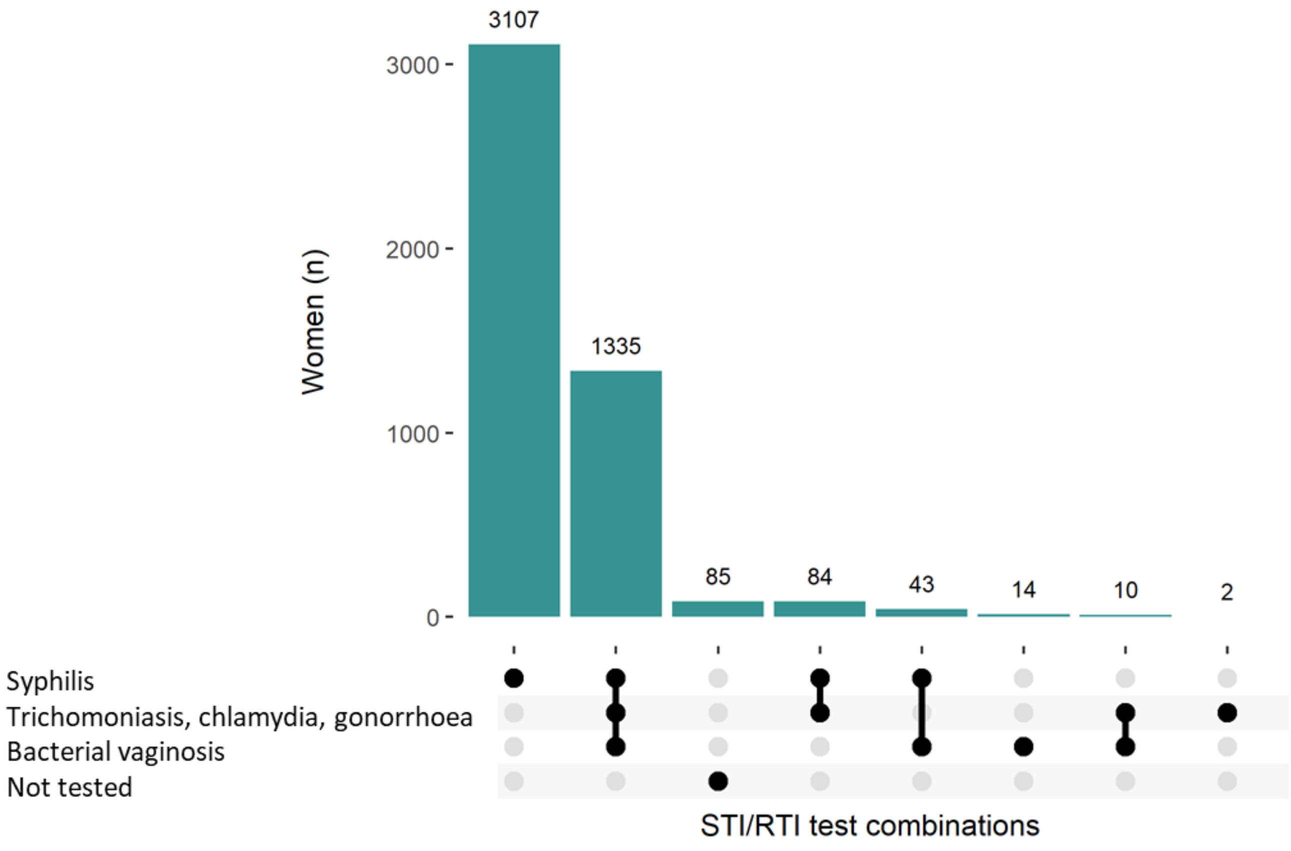


**Figure 1: Unique STI/RTI test combinations and number tested**

| Number of STI/RTIs tested for | Women (n) | Percentage (%) |
|---|---|---|
| 1 | 3,121 | 67.9 |
| 2 | 43 | 0.9 |
| 3 | 2 | 0.04 |
| 4 | 94 | 2.1 |
| 5 | 1,335 | 29.1 |
| Total | 4,595 | 100.0 |

**Table 2: Total number of STI/RTI tests done per woman**

**Table 3: Bacterial vaginosis and syphilis infection results**

| Infection | Categorical Outcome | N Positive / N Tested | Percentage (%) |
|---|---|---|---|
| Bacterial Vaginosis (Nugent score) | **7-10 (positive)** | 399/1402 | 28.5 |
| | **4-6 (intermediate)** | 137/1402 | 9.8 |
| | **0-3 (negative)** | 866/1402 | 61.8 |
| **Syphilis (RPR titre)** | **≥1:8** | 34/79 | 43.0 |
| | **≤1:4** | 45 /79 | 57.0 |

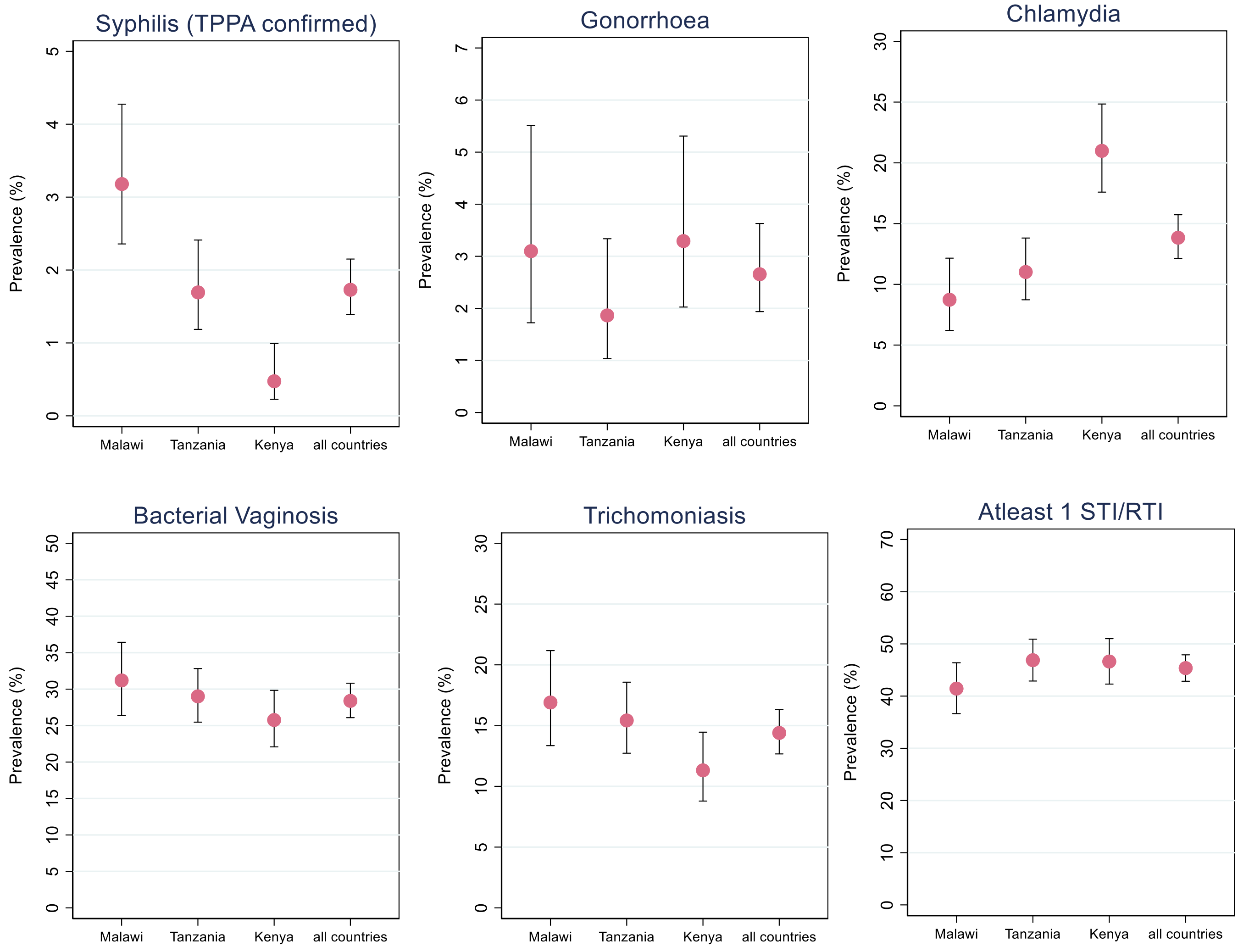


**Figure 2: Prevalence of each individual infection and prevalence of at least one of chlamydia, gonorrhoea, trichomoniasis, or bacterial vaginosis**

**Table 4: Logistic regression models for association of risk factors with syphilis prevalence (all models controlled for country and site)**

| | | N with STI/N tested (prevalence (%)) | Univariable | | Multivariable 1 | | Multivariable 2 | |
|---|---|---|---|---|---|---|---|---|
| | | | PR (95%CI) | p-value | PR (95%CI) | p-value | PR (95%CI) | p-value |
| **Maternal age** | **<25 years** | 38/2,423 (1.6) | ref | | ref | | 1.4 (0.4, 4.9) | 0.578 |
| | **≥25 years** | 41/2,135 (1.9) | 1.70 (1.09, 2.66) | 0.020 | 0.79 (0.21, 2.97) | 0.731 | ref | |
| **Gravidity** | **Paucigravid** | 32/2,523 (1.3) | ref | | ref | | 0.5 (0.2, 1.7) | 0.274 |
| | **Multigravid** | 47/2,003 (2.3) | 1.18 (0.76, 1.83) | 0.465 | 1.91 (0.53, 6.90) | 0.325 | ref | |
| **Marital status** | **Single** | 4/566 (0.7) | ref | | ref | | [redacted] | |
| | **Married** | 75/4,001 (1.9) | 1.74 (0.62, 4.90) | 0.296 | 0.70 (0.18, 2.74) | 0.609 | | |
| **SES tercile** | **low** | 44/1,516 (2.9) | ref | | ref | | 8.8 (1.0, 76.1) | 0.048 |
| | **medium** | 26/1,525 (1.7) | 1.01 (0.61, 1.67) | 0.964 | 1.33 (0.47, 3.76) | 0.585 | 8.8 (1.1, 68.1) | 0.037 |
| | **high** | 9/1,528 (0.6) | 0.36 (0.16, 0.80) | 0.012 | 0.16 (0.02, 1.45) | 0.103 | ref | |
| **Maternal education** | **none/primary** | 65/2,962 (2.2) | ref | | ref | | [redacted] | |
| | **Secondary** | 14/1,598 (0.9) | 0.50 (0.28, 0.90) | 0.021 | 0.87 (0.31, 2.48) | 0.795 | | |
| **Residency** | **Rural** | 67/3,351 (2.0) | ref | | Ref | | | |
| | **Semi-/Urban** | 12/1,216 (1.0) | 0.47 (0.25, 0.88) | 0.019 | 0.93 (0.30, 2.84) | 0.894 | | |
| **Anaemia** | **No** | 45/2,535 (1.8) | ref | | ref | | | |
| | **Yes** | 34/2,017 (1.7) | 1.0 (0.65, 1.56) | 0.983 | 2.27 (1.02, 5.09) | 0.046 | | |
| **BV** | **Negative** | 9/988 (0.9) | ref | | ref | | ref | |
| | **Positive** | 14/390 (3.6) | 3.77 (1.66, 8.57) | 0.002 | 3.73 (1.68, 8.31) | 0.001 | 3.7 (1.6, 8.4) | 0.002 |
| Multivariable 1: controlling for all variables | | | | | | | | |
| Multivariable 2: controlling just for variables relevant in step-wise method (change PR by 10%). NB in some cases, reference groups changed for ease of interpretation | | | | | | | | |

**Table 5: Logistic regression models for association of risk factors with chlamydia prevalence (all models controlled for country and site)**

| | | N with STI/N tested (prevalence (%)) | Univariable | | Multivariable 1 | | Multivariable 2 | |
|---|---|---|---|---|---|---|---|---|
| | | | PR (95%CI) | p-value | PR (95%CI) | p-value | PR (95%CI) | p-value |
| **Maternal age** | **<25 years** | 124/763 (16.3) | ref | | ref | | 1.0 (0.7, 1.4) | 0.955 |
| | **≥25 years** | 74/668 (11.1) | 0.73 (0.55, 0.96) | 0.022 | 0.97 (0.69, 1.37) | 0.852 | ref | |
| **Gravidity** | **Paucigravid** | 137/783 (17.5) | ref | | ref | | 1.7 (1.2, 2.4) | 0.002 |
| | **Multigravid** | 61/642 (9.5) | 0.58 (0.44, 0.77) | 0.000 | 0.66 (0.46, 0.93) | 0.018 | ref | |
| **Marital status** | **Single** | 36/176 (20.5) | ref | | ref | | | |
| | **Married** | 162/1254 (12.9) | 0.83 (0.59, 1.16) | 0.268 | 1.08 (0.75, 1.57) | 0.670 | | |
| **SES tercile** | **low** | 38/466 (8.2) | ref | | ref | | | |
| | **medium** | 82/485 (16.9) | 1.67 (1.08, 2.59) | 0.021 | 1.54 (0.97, 2.45) | 0.070 | | |
| | **high** | 78/480 (16.3) | 1.64 (1.04, 2.60) | 0.034 | 1.48 (0.86, 2.55) | 0.156 | | |
| **Maternal education** | **none/primary** | 114/900 (12.7) | ref | | ref | | | |
| | **Secondary** | 83/528 (15.7) | 1.14 (0.86, 1.51) | 0.353 | 0.91 (0.66, 1.26) | 0.573 | | |
| **Residency** | **Rural** | 141/1045 (13.5) | ref | | ref | | | |
| | **Semi-/Urban** | 57/385 (14.8) | 1.49 (1.05, 2.11) | 0.025 | 1.40 (0.96, 2.04) | 0.084 | | |
| **Anaemia** | **No** | 94/777 (12.1) | ref | | ref | | | |
| | **Yes** | 104/651 (16.0) | 1.25 (0.97, 1.62) | 0.085 | 1.30 (0.99, 1.70) | 0.055 | | |
| **BV** | **Negative** | 120/961 (12.5) | ref | | ref | | | |
| | **Positive** | 73/384 (19.0) | 1.55 (1.19, 2.02) | 0.001 | 1.51 (1.16, 1.96) | 0.002 | | |
| Multivariable 1: controlling for all variables | | | | | | | | |
| Multivariable 2: controlling just for variables relevant in step-wise method (change PR by 10%). NB in some cases, reference groups changed for ease of interpretation | | | | | | | | |

**Table 6: Logistic regression models for association of risk factors with bacterial vaginosis prevalence (all models controlled for country and site)**

| | | N with STI/N tested (prevalence (%)) | Univariable | | Multivariable 1 | | Multivariable 2 | |
|---|---|---|---|---|---|---|---|---|
| | | | PR (95%CI) | p-value | PR (95%CI) | p-value | PR (95%CI) | p-value |
| **Maternal age** | **<25 years** | 222/744 (29.8) | ref | | ref | | 1.0 (0.8, 1.2) | 0.752 |
| | **≥25 yrs** | 177/658 (26.9) | 0.89 (0.75, 1.05) | 0.156 | 1.03 (0.83, 1.29) | 0.767 | ref | |
| **Gravidity** | **Paucigravid** | 241/774 (31.1) | ref | | ref | | 1.3 (1.1, 1.6) | 0.018 |
| | **Multigravid** | 156/623 (25.0) | 0.79 (0.66, 0.93) | 0.006 | 0.77 (0.62, 0.97) | 0.025 | | |
| **Marital status** | **Single** | 54/174 (31.0) | ref | | ref | | | |
| | **Married** | 344/1227 (28.0) | 0.86 (0.67, 1.10) | 0.225 | 0.94 (0.72, 1.22) | 0.630 | | |
| **SES tercile** | **low** | 133/440 (30.2) | ref | | ref | | | |
| | **medium** | 129/474 (27.2) | 0.97 (0.77, 1.22) | 0.786 | 0.94 (0.74, 1.19) | 0.610 | | |
| | **high** | 137/488 (28.1) | 1.00 (0.79, 1.26) | 0.997 | 0.97 (0.74, 1.28) | 0.827 | | |
| **Maternal education** | **none/primary** | 245/869 (28.2) | ref | | ref | | | |
| | **Secondary** | 153/530 (28.9) | 1.07 (0.90, 1.28) | 0.439 | 1.02 (0.83, 1.26) | 0.815 | | |
| **Residency** | **Rural** | 294/1026 (28.7) | ref | | ref | | | |
| | **Semi-/Urban** | 104/375 (27.7) | 0.95 (0.76, 1.20) | 0.670 | 0.93 (0.73, 1.19) | 0.574 | | |
| **Anaemia** | **No** | 220/766 (28.7) | ref | | ref | | | |
| | **Yes** | 177/633 (28.0) | 0.99 (0.83, 1.17) | 0.893 | 0.96 (0.81, 1.14) | 0.630 | | |
| Multivariable 1: controlling for all variables | | | | | | | | |
| Multivariable 2: controlling just for variables relevant in step-wise method (change PR by 10%). NB in some cases, reference groups changed for ease of interpretation | | | | | | | | |

**Table 7: Logistic regression models for association of risk factors with gonorrhoea prevalence (all models controlled for country and site)**

| | | N with STI/N tested (prevalence (%)) | Univariable | | Multivariable 1 | | Multivariable 2 | |
|---|---|---|---|---|---|---|---|---|
| | | | PR (95%CI) | p-value | PR (95%CI) | p-value | PR (95%CI) | p-value |
| **Maternal age** | **<25 years** | 28/763 (3.7) | ref | | ref | | 1.6 (0.7, 3.3) | 0.237 |
| | **≥25 yrs** | 10/668 (1.5) | 0.44 (0.21, 0.93) | 0.031 | 0.66 (0.31, 1.39) | 0.274 | ref | |
| **Gravidity** | **Paucigravid** | 29/783 (3.7) | ref | | ref | | 2.5 (1.2, 5.4) | 0.018 |
| | **Multigravid** | 9/642 (1.4) | 0.39 (0.18, 0.81) | 0.012 | 0.40 (0.19, 0.88) | 0.022 | ref | |
| **Marital status** | **Single** | 8/176 (4.5) | ref | | ref | | | |
| | **Married** | 30/1254 (2.4) | 0.64 (0.30, 1.34) | 0.231 | 0.88 (0.41, 1.91) | 0.746 | | |
| **SES tercile** | **low** | 14/466 (3.0) | ref | | ref | | 0.9 (0.3, 2.6) | 0.876 |
| | **medium** | 11/485 (2.3) | 0.58 (0.20, 1.72) | 0.327 | 0.50 (0.17, 1.47) | 0.209 | 0.5 (0.2, 1.0) | 0.054 |
| | **high** | 13/480 (2.7) | 0.79 (0.28, 2.20) | 0.653 | 1.06 (0.35, 3.23) | 0.923 | ref | |
| **Maternal education** | **none/primary** | 27/900 (3.0) | ref | | ref | | 2.4 (1.2, 5.1) | 0.016 |
| | **Secondary** | 11/528 (2.1) | 0.67 (0.32, 1.39) | 0.281 | 0.40 (0.20, 0.82) | 0.012 | ref | |
| **Residency** | **Rural** | 29/1045 (2.8) | ref | | ref | | | |
| | **Semi-/Urban** | 9/385 (2.3) | 1.07 (0.50, 2.25) | 0.869 | 1.27 (0.54, 2.99) | 0.579 | | |
| **Anaemia** | **No** | 18/777 (2.3) | ref | | ref | | | |
| | **Yes** | 20/651 (3.1) | 1.43 (0.78, 2.63) | 0.245 | 1.17 (0.63, 2.17) | 0.608 | | |
| **BV** | **Negative** | 21/961 (2.2) | ref | | ref | | ref | |
| | **Positive** | 16/384 (4.2) | 2.03 (1.06, 3.89) | 0.032 | 1.79 (0.94, 3.39) | 0.075 | 1.8 (1.0, 3.4) | 0.074 |
| Multivariable 1: controlling for all variables | | | | | | | | |
| Multivariable 2: controlling just for variables relevant in step-wise method (change PR by 10%) | | | | | | | | |

NB reference groups changed in main paper

**Table 8: Logistic regression models for association of risk factors with trichomoniasis prevalence (all models controlled for country and site)**

| | | N with STI/N tested (prevalence (%)) | Univariable | | Multivariable 1 | | Multivariable 2 | |
|---|---|---|---|---|---|---|---|---|
| | | | PR (95%CI) | p-value | PR (95%CI) | p-value | PR (95%CI) | p-value |
| **Maternal age** | **<25 years** | 115/763 (15.1) | ref | | ref | | ref | |
| | **≥25 years** | 92/668 (13.8) | 0.90 (0.69, 1.16) | 0.405 | 0.83 (0.59, 1.17) | 0.278 | 0.90 (0.70, 1.17) | 0.435 |
| **Gravidity** | **Paucigravid** | 113/783 (14.4) | ref | | ref | | | |
| | **Multigravid** | 94/642 (14.6) | 1.01 (0.78, 1.30) | 0.954 | 1.03 (0.73, 1.46) | 0.860 | | |
| **Marital status** | **Single** | 30/176 (17.0) | ref | | ref | | | |
| | **Married** | 177/1254 (14.1) | 0.75 (0.52, 1.08) | 0.118 | 0.79 (0.54, 1.17) | 0.237 | | |
| **SES tercile** | **low** | 85/466 (18.2) | ref | | ref | | | |
| | **medium** | 60/485 (12.4) | 0.76 (0.54, 1.07) | 0.114 | 0.85 (0.59, 1.21) | 0.365 | | |
| | **high** | 62/480 (12.9) | 0.78 (0.54, 1.11) | 0.165 | 1.00 (0.66, 1.52) | 0.986 | | |
| **Maternal education** | **none/primary** | 145/900 (16.1) | ref | | ref | | | |
| | **Secondary** | 62/528 (11.7) | 0.75 (0.56, 1.00) | 0.047 | 0.76 (0.54, 1.06) | 0.109 | | |
| **Residency** | **Rural** | 158/1045 (15.1) | ref | | ref | | | |
| | **Semi-/Urban** | 49/385 (12.7) | 0.74 (0.52, 1.06) | 0.097 | 0.77 (0.53, 1.13) | 0.183 | | |
| **Anaemia** | **No** | 113/777 (14.5) | ref | | ref | | | |
| | **Yes** | 94/651 (14.4) | 1.09 (0.84, 1.41) | 0.524 | 1.03 (0.78, 1.35) | 0.848 | | |
| **BV** | **Negative** | 145/961 (15.1) | ref | | ref | | | |
| | **Positive** | 52/384 (13.5) | 0.89 (0.66, 1.20) | 0.439 | 0.88 (0.66, 1.18) | 0.403 | | |
| Multivariable 1: controlling for all variables | | | | | | | | |
| Multivariable 2: controlling just for variables relevant in step-wise method (change PR by 10%) | | | | | | | | |

**Table 9: Logistic regression models for association of risk factors with at least one of CT/NG/TV/BV prevalence (all models controlled for country and site)**

| | | N with STI/N tested (prevalence (%)) | Univariable | | Multivariable 1 | | Multivariable 2 | |
|---|---|---|---|---|---|---|---|---|
| | | | PR (95%CI) | p-value | PR (95%CI) | p-value | PR (95%CI) | p-value |
| **Maternal age** | **<25 years** | 385/797 (48.3) | ref | | ref | | ref | |
| | **≥25 years** | 298/691 (43.1) | 0.88 (0.79, 0.99) | 0.031 | 1.03 (0.83, 1.29) | 0.767 | 0.95 (0.82, 1.09) | 0.446 |
| **Gravidity** | **Paucigravid** | 402/821 (49.0) | ref | | ref | | ref | |
| | **Multigravid** | 279/661 (42.2) | 0.86 (0.76, 0.96) | 0.007 | 0.77 (0.62, 0.97) | 0.025 | 0.88 (0.76, 1.02) | 0.090 |
| **Marital status** | **Single** | 96/182 (52.7) | ref | | ref | | | |
| | **Married** | 586/1305 (44.9) | 0.88 (0.75, 1.03) | 0.102 | 0.94 (0.72, 1.22) | 0.630 | | |
| **SES tercile** | **low** | 225/483 (46.6) | ref | | ref | | | |
| | **medium** | 230/503 (45.7) | 0.93 (0.80, 1.09) | 0.386 | 0.94 (0.74, 1.19) | 0.610 | | |
| | **high** | 228/502 (45.4) | 0.93 (0.79, 1.09) | 0.388 | 0.97 (0.74, 1.28) | 0.827 | | |
| **Maternal education** | **none/primary** | 429/931 (46.1) | ref | | ref | | | |
| | **Secondary** | 252/554 (45.5) | 1.00 (0.89, 1.13) | 0.988 | 1.02 (0.83, 1.26) | 0.815 | | |
| **Residency** | **Rural** | 506/1083 (46.7) | ref | | ref | | | |
| | **Semi-/Urban** | 176/404 (43.6) | 0.97 (0.83, 1.13) | 0.681 | 0.93 (0.73, 1.19) | 0.574 | | |
| **Anaemia** | **No** | 367/810 (45.3) | ref | | ref | | | |
| | **Yes** | 314/675 (46.5) | 1.03 (0.92, 1.16) | 0.551 | 0.96 (0.81, 1.14) | 0.630 | | |
| Multivariable 1: controlling for all variables | | | | | | | | |
| Multivariable 2: controlling just for variables relevant in step-wise method (change PR by 10%) | | | | | | | | |

**Table 10: Proportion of women positive with each individual STI/RTI who are co-infected with each of the other four STI/RTIs**

| Among women positive for… | …proportion co-infected with: n/N, % (95% CI) | | | | |
|---|---|---|---|---|---|
| | **Bacterial vaginosis** | **Trichomoniasis** | **Gonorrhoea** | **Chlamydia** | **Syphilis** |
| **Bacterial vaginosis** | | 52/384, 13.5 (10.5, 17.4) | 16/384, 4.2 (2.6, 6.7) | 73/384, 19.0 (15.4, 23.3) | 14/391, 3.6 (2.1, 6.0) |
| **Trichomoniasis** | 52/197, 26.4 (20.7, 33.1) | | 8/207, 3.9 (1.9, 7.6) | 34/207, 16.4 (11.9, 22.2) | 8/205, 3.9 (2.0, 7.7) |
| **Gonorrhoea** | 16/37, 43.2 (27.8, 60.1) | 8/38, 21.1 (10.5, 37.6) | | 12/38, 31.6 (18.4, 48.6) | 2/38, 5.3 (1.2, 19.8) |
| **Chlamydia** | 73/193, 37.8 (31.2, 44.9) | 34/198, 17.2 (12.5, 23.1) | 12/198, 6.1 (3.5, 10.4) | | 4/196, 2.0 (0.8, 5.4) |
| **Syphilis** | 14/23, 60.8 (38.6, 79.4) | 8/24, 33.3 (16.7, 55.5) | 2/24, 8.3 (1.9, 30.2) | 4/24, 16.7 (5.9, 38.9) | |

**Table 11: Multinomial logistic regression models for association of risk factors with the three most common co-infection combinations**

| Co-infection | | Gravidity | RRR (95% CI) | P-value |
|---|---|---|---|---|
| BV & TV (reference group: negative for BV & TV) | BV only | Pauci- | 1.18 (0.83, 1.67) | 0.351 |
| | | Multi- | ref | |
| | TV only | Pauci- | 0.72 (0.45, 1.78) | 0.194 |
| | | Multi- | ref | |
| | BV & TV | Pauci- | 2.58 (1.16, 5.73) | 0.020 |
| | | Multi- | ref | |
| BV & CT (reference group: negative for BV & CT) | BV only | Pauci- | 1.22 (0.86, 1.74) | 0.264 |
| | | Multi- | ref | |
| | CT only | Pauci- | 1.41 (0.83, 2.39) | 0.200 |
| | | Multi- | ref | |
| | BV & CT | Pauci- | 2.86 (1.43, 5.70) | 0.003 |
| | | Multi- | ref | |
| TV & CT (reference group: negative for CT & TV) | TV only | Pauci- | 0.86 (0.55, 1.34) | 0.500 |
| | | Multi- | ref | |
| | CT only | Pauci- | 1.74 (1.10, 2.75) | 0.018 |
| | | Multi- | ref | |
| | TV & CT | Pauci- | 1.90 (0.70, 5.17) | 0.204 |
| | | Multi- | ref | |
| NB. All models control for maternal age, maternal education, marital status, SES, residency (urban/rural), anaemic status, country, and site. BV= bacterial vaginosis, TV= trichomoniasis, CT = chlamydia | | | | |

**Table 12: Association between each STI/RTIs pair (crude and adjusted):**

| Exposure | Outcome<br>Odds Ratio (95% CI), p-value | | | | |
|---|---|---|---|---|---|
| | **Bacterial vaginosis** | **Trichomoniasis** | **Gonorrhoea** | **Chlamydia** | **Syphilis** |
| **Bacterial vaginosis** | | C: 0.9 (0.7, 1.2), p=0.471 | C: 1.9 (1.0, 3.6), p=0.048 | C: 1.5 (1.2, 2.0), p=0.002 | C: 3.9 (1.7, 9.0), p=0.001 |
| | | A: 0.9 (0.7, 1.2), p=0.403 | A: 1.8 (0.9, 3.4), p=0.075 | A: 1.5 (1.2, 2.0), p=0.002* | A: 3.7 (1.6, 8.6), p=0.002* |
| **Trichomoniasis** | C: 0.9 (0.7, 1.2), p=0.475 | | C: 1.6 (0.7, 3.4), p=0.244 | C: 1.2 (0.9, 1.7), p=0.239 | C: 3.1 (1.3, 6.8), p=0.011 |
| | A: 0.9 (0.7, 1.2), p=0.402 | | A: 1.4 (0.6, 3.2), p=0.378 | A: 1.3 (1.0, 1.9), p=0.076 | A: 3.0 (1.4, 6.8), p=0.007 |
| **Gonorrhoea** | C: 1.5 (1.1, 2.2), p=0.026 | C: 1.5 (0.8, 2.8), p=0.227 | | C: 2.4 (1.5, 2.8), p=0.001 | C: 3.3 (0.8, 12.6), p=0.097 |
| | A: 1.5 (1.0, 2.3), p=0.033* | A: 1.4 (0.7, 2.7), p=.0321 | | A: 2.0 (1.2, 3.2), p=0.006* | A: 2.7 (0.8, 9.1), p=0.106 |
| **Chlamydia** | C: 1.4 (1.1, 1.7), p=0.001 | C: 1.2 (0.9, 1.7), p=0.238 | C: 2.9 (1.5, 5.6), p=0.002 | | C: 1.2 (0.4, 3.6), p=0.683 |
| | A: 1.4 (1.1, 1.7), p=0.001* | A: 1.4 (1.0, 1.9), p=0.068 | A: 2.5 (1.2, 5.0), p=0.012* | | A: 1.6 (0.6, 4.5), p=0.365 |
| **Syphilis** | C: 2.2 (1.6, 3.1), p=<0.001 | C: 2.4 (1.3, 4.2), p=0.004 | C: 3.2 (0.8, 12.7), p=0.093 | C: 1.2 (0.5, 3.0), p=0.678 | |
| | A: 2.2 (1.5, 3.1), p=<0.001* | A: 2.3 (1.2, 4.2), p=0.008* | A: 2.6 (0.7, 10.4), p=0.192 | A: 1.6 (0.7, 3.8), p=0.290 | |

**C: Crude, A: Adjusted – controlling for all possible confounders.**
***adjusted model significant at p<0.05**

**Table 13: Prevalence of malaria and curable STI/RTI co-infection**

| Co-infection status | Positive (n) | Tested (n) | Prevalence (%) | (95% CI) |
|---|---|---|---|---|
| **Malaria and:** | | | | |
| **Syphilis** | 8 | 4,129 | 0.2 | 0.1, 0.4 |
| **Chlamydia** | 30 | 1,346 | 2.2 | 1.6, 3.2 |
| **Gonorrhoea** | 9 | 1,346 | 0.7 | 0.3, 1.3 |
| **Trichomoniasis** | 21 | 1,346 | 1.6 | 1.0, 2.4 |
| **Bacterial vaginosis** | 55 | 1,317 | 4.2 | 3.2, 5.4 |
| **any STI/RTI** | 94 | 1,401 | 6.7 | 5.0, 8.9 |
| **1 STI/RTI** | 82 | 1,401 | 5.9 | 4.7, 7.2 |
| **2 STI/RTIs** | 11 | 1,401 | 0.8 | 0.4, 1.4 |
| **3 STI/RTIs** | 5 | 1,401 | 0.4 | 0.1, 0.9 |
| **4 STI/RTIs** | 1 | 1,401 | 0.1 | 0.0, 0.5 |

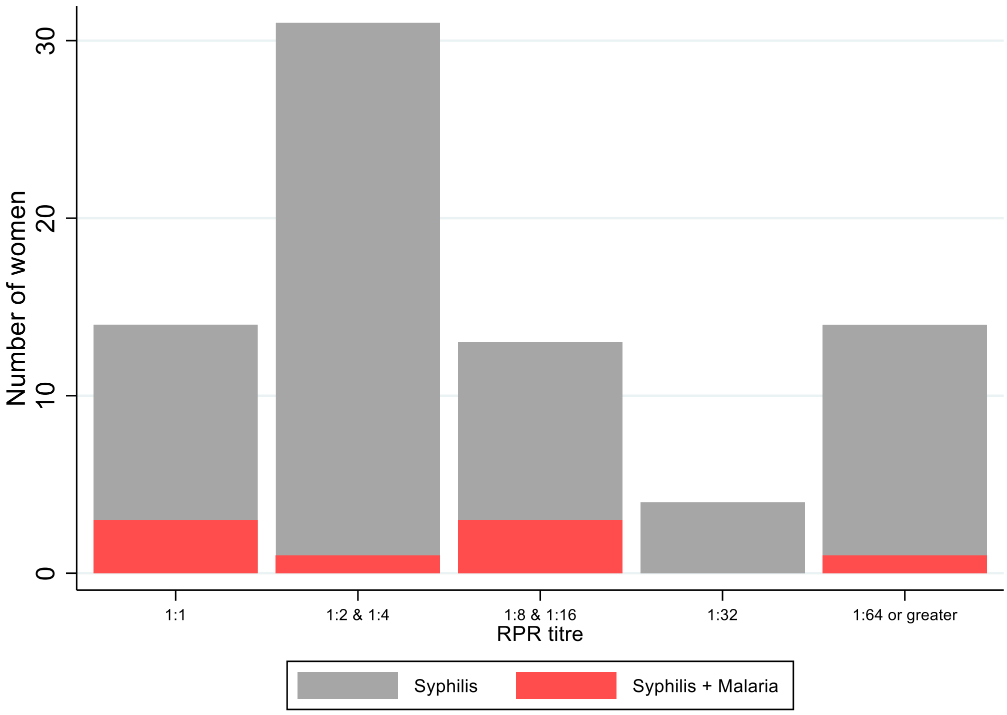


**Figure 3: Number of TPPA confirmed syphilis cases by RPR titre (grey bar) and number co-infected with malaria (red bar)**

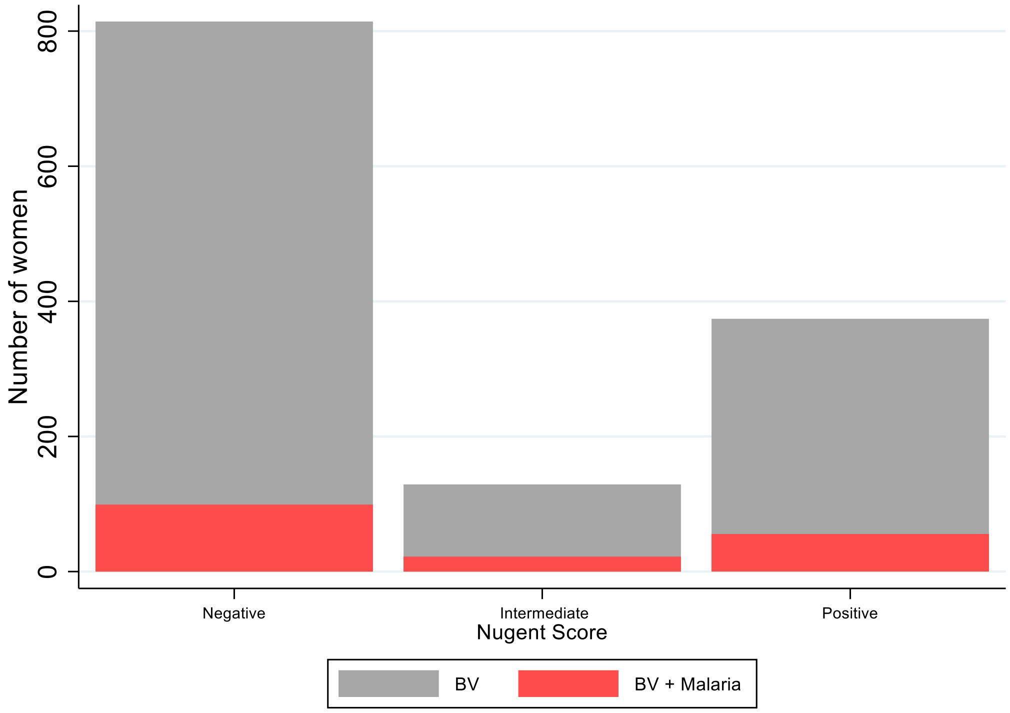


**Figure 4: Number of BV cases by categorical Nugent score (grey bar) and number co-infected with malaria (red bar)**

**Table 14: Number and proportion of women co-infected with malaria by categorical BV and syphilis status**

| | | Malaria co-infected / total STI/RTI positive | Malaria prevalence % (95% CI) |
|---|---|---|---|
| BV | Negative (Nugent score 0-3) | 99/814 | 12.16 (10.09, 14.60) |
| | Intermediate (Nugent score 4-6) | 22/129 | 17.05 (11.49, 24.56) |
| | Positive (Nugent score ≥7) | 55/374 | 14.71 (11.46, 18.67) |
| Syphilis | 1:1 | 3/14 | 21.43 (6.88, 50.15) |
| | 1:2 & 1:4 | 1/31 | 3.23 (4.32, 20.38) |
| | 1:8 & 1:16 | 3/13 | 23.08 (7.42, 52.90) |
| | 1:32 | 0/4 | 0.00 |
| | >1:32 | 1/14 | 7.14 (9.51, 38.13) |

| Co-infection Combination | Infection Category | Risk Factor | RRR (95% CI) | P-value |
|---|---|---|---|---|
| Malaria & Bacterial Vaginosis (reference group: negative for both) | Malaria only | Rural | Ref | 0.301 |
| | | Semi-/Urban | 0.7 (0.4, 1.4) | |
| | | No anaemia | Ref | <0.001 |
| | | Anaemia | 3.7 (2.4, 5.9) | |
| | Bacterial Vaginosis only | Rural | Ref | 0.931 |
| | | Semi-/Urban | 1.0 (0.70, 1.5) | |
| | | No anaemia | Ref | 0.956 |
| | | Anaemia | 1.0 (0.8, 1.3) | |
| | **Malaria & Bacterial Vaginosis** | Rural | Ref | 0.036 |
| | | Semi-/Urban | 0.3 (0.1, 0.9) | |
| | | No anaemia | Ref | 0.011 |
| | | Anaemia | 2.2 (1.2, 4.1) | |
| Malaria & Chlamydia (reference group: negative for both) | Malaria only | Single | Ref | 0.372 |
| | | Married | 0.8 (0.4, 1.4) | |
| | | No anaemia | Ref | <0.001 |
| | | Anaemia | 3.1 (2.1, 4.6) | |
| | Chlamydia only | Single | Ref | 0.524 |
| | | Married | 1.2 (0.7, 2.0) | |
| | | No anaemia | Ref | 0.224 |
| | | Anaemia | 1.2 (0.9, 1.8) | |
| | **Malaria & Chlamydia** | Single | Ref | 0.023 |
| | | Married | 0.3 (0.1, 0.9) | |
| | | No anaemia | Ref | <0.001 |
| | | Anaemia | 7.7 (2.5, 23.7) | |
| Malaria + Trichomoniasis (reference group: negative for both) | Malaria only | Single | Ref | 0.163 |
| | | Married | 0.7 (0.4, 1.2) | |
| | | No anaemia | Ref | <0.001 |
| | | Anaemia | 3.4 (2.3, 5.0) | |
| | Trichomoniasis only | Single | Ref | 0.426 |
| | | Married | 0.8 (0.5, 1.4) | |
| | | No anaemia | Ref | 0.737 |
| | | Anaemia | 1.1 (0.8, 1.5) | |
| | **Malaria & Trichomoniasis** | Single | Ref | 0.047 |
| | | Married | 0.3 (0.1, 1.0) | |
| | | No anaemia | Ref | 0.013 |
| | | Anaemia | 3.5 (1.3, 9.6) | |
| NB. All models control for all other variables | | | | |

**Table 15: Multinomial logistic regression models for association of risk factors with malaria and bacterial vaginosis/chlamydia/trichomoniasis co-infection.**
